# Multi-omic insights from a multi-ancestry genome-wide meta-analysis of ankylosing spondylitis reveal novel pathways of disease susceptibility

**DOI:** 10.21203/rs.3.rs-6917334/v1

**Published:** 2025-07-14

**Authors:** Matthew Brown, Zhixiu Li, Xin Wu, Nicholas Harvey, Jose Garrido-Mesa, Xiaobing Wang, Zhihao Xu, Geng Wang, Helena Marzo-Ortega, Dennis McGonagle, Ann Morgan, Nurullah Akkoc, Gokce Kenar Artin, Gary Macfarlane, Gareth Jones, Linda Bradbury, Paul Leo, Kate Zimmerman, Emma Duncan, Julia Brown, Tony Merriman, Simon Stebbings, Mahdi Mahmoudi, Ahmadreza Jamshidi, Elham Farhadi, Nigil Haroon, Robert Inman, Maxime Breban, Gensler Lianne, Michael Ward, B. Wordsworth, Michael Weisman, David Evans, Tony Kenna, Tae-Hwan Kim, John Reveille, Huji Xu

**Affiliations:** King’s College London; School of Public Health and Emergency Management, Southern University of Science and Technology; Department of Rhuematology and Immunology, Shanghai Changzeng Hospital, The Second Military Medical University, China.; Department of Medical and Molecular Genetics, Faculty of Life Sciences and Medicine, King’s College London; Unidad investigación clínica de cáncer de pulmón, H12O-CNIO, Instituto de Investigación Hospital 12 de Octubre/Centro Nacional de Investigaciones Oncológica; Department of Rheumatology and Immunology, National key laboratory for Immunity and Inflammation, Shanghai Changzheng Hospital, Naval Medical University; School of Public Health and Emergency Management, Southern University of Science and Technology; The University of Queensland; Leeds Institute of Rheumatic and Musculoskeletal Medicine, University of Leeds; Leeds Institute of Rheumatic and Musculoskeletal Medicine, Chapel Allerton Hospital, University of Leeds; Leeds University; Dokuz Eylül University; School of Medicine, Division of Rheumatology, Dokuz Eylül University; School of Medicine and Dentistry, University of Aberdeen; University of Aberdeen; Queensland University of Technology; QUT; School of Biomedical Sciences, Queensland University of Technology; University of Queensland; College of Medicine and Dentistry, James Cook University; University of Alabama at Birmingham; Department of Medicine, Dunedin School of Medicine, University of Otago; Research Center for Chronic Inflammatory Diseases, Tehran University of Medical Sciences and Rheumatology Research Center, Tehran University of Medical Sciences; Research Center for Chronic Inflammatory Diseases, Tehran University of Medical Sciences and Rheumatology Research Center, Tehran University of Medical Sciences; Research Center for Chronic Inflammatory Diseases, Tehran University of Medical Sciences and Rheumatology Research Center, Tehran University of Medical Sciences; Division of Rheumatology, Department of Medicine, University Health Network, University of Toronto, Schroeder Arthritis Institute; Division of Rheumatology, Department of Medicine, University Health Network, University of Toronto, Schroeder Arthritis Institute; UMR1173, Université Paris Saclay, Université de Versailles St Quentin en Yvelines, Inserm, Infection et Inflammation; University of California, San Francisco; National Institutes of Health; Botnar Research Centre; Stanford University; University of Queensland; Queensland University of Technology; Hanyang University Hospital for Rheumatic Diseases; Memorial Hermann Texas Medical Centre; Department of Rheumatology and Immunology, National Key Laboratory for Immunity and Inflammation, Shanghai Changzheng Hospital, Naval Medical University

## Abstract

We report the largest genome-wide association study meta-analysis in ankylosing spondylitis (AS) to date (25,645 cases, 71,224 controls), identifying 27 novel loci and 86 independent genetic associations. Variations in *FUT2* (non-secretor status) and *ABO* (blood group A) increase AS risk, with Mendelian randomisation (MR) linking non-secretor status to increased AS risk from reduced gut carriage of *Ruminococcus torques*. Associations with three telomerase maintenance genes (*TERT, TERC*, *RTEL1*), and MR analysis, suggest increased telomere length causally increases AS susceptibility. Fine-mapping prioritised likely causal variants at multiple loci. Transcriptome- and proteome-wide association studies implicated 644 genes, highlighting immune-related pathways. Lower genetically-determined IL-6 and IL-12, and similar IL-23, levels were found in AS cases, offering a genetic explanation for the failure of IL-6, IL-12, and IL-23 inhibition in AS treatment. Finally, multi-omic analyses showed chromosome 2p15 association acts via reduced *B3GNT2* expression. These findings deepen understanding of AS pathogenesis, highlighting new pathways and therapeutic opportunities.

## Introduction

Ankylosing spondylitis (AS) (also known as radiographic axial spondyloarthritis) is a common inflammatory condition with a prevalence of 0.1–0.7% in most global populations ^1,2^, affecting primarily joints of the spine and pelvis. Extramusculoskeletal manifestations of the condition include acute anterior uveitis (affecting ultimately over 50% of patients during their lifetime ^3^); terminal ileitis (found subclinically in approximately 60% of patients) ^4^; Crohn’s disease and ulcerative colitis (up to 17% of patients ^5^); psoriasis (up to 14% of patients ^6^); and, rarely, aortitis, pneumonitis, and IgA nephropathy. AS is associated with significant morbidity and early mortality, with a standardised mortality rate of 1.37 ^3^. AS typically develops between 18 and 45 years of age and is more common in males (male:female gender ratio approximately 2:1). The condition runs strongly in families (sibling recurrence risk ratio 82) ^7^, and has high heritability estimated in twins at > 90% ^8,9^. The major locus is the major histocompatibility complex (MHC) where HLA-B27, the major risk allele, is encoded. MHC associations with the disease are complex, however, and involve other risk and protective HLA-B alleles and other HLA Class I and Class II associations ^10–12^. Although more than 80% of AS cases carry HLA-B27, only approximately 1–5% of carriers develop AS, which likely reflects the influence of non-MHC genetic associations. To date, at least 116 independent genetic associations have been robustly identified as associated with the disease, contributing approximately 28% of the overall genetic risk ^10,13–18^.

With the goal of advancing understanding of the genetic aetiology of AS, we report here the findings of a genome-wide association study involving 25,645 cases and 71,224 controls, meta-analysing data from cohorts of European (EUR), East Asian (EAS), Iranian, and Turkish ancestries. This study identifies 27 previously unreported genome-wide significant (GWS, P < 5×10^−8^) loci and demonstrates strong genetic correlation between different ancestral groups. Additional associations are noted through transcriptome-wide association studies (TWAS) (n = 438) and proteome-wide association studies (PWAS) (n = 178), providing further insight into the genetic factors involved in AS pathogenesis.

## Results

### Primary association findings

Following all quality control filters, the EUR cohort consisted of 15,913 cases and 59,769 controls, the EAS cohort 8,372 cases and 9,754 controls, the Iranian 420 cases and 755 controls, and the Turkish cohort 940 cases and 946 controls (in total 25,645 cases and 71,224 controls) (Table S1). Excluding the MHC, the genomic inflation factor in the combined dataset was 1.15 (l_1000_ = 1.004), the EUR cohort 1.178 (l_1000_ = 1.007), the EAS cohort 1.105 (l_1000_ = 1.012), the Turkish cohort 1.028 (l_1000_ = 1.030) and Iranian cohort 1.021 (l_1000_ = 1.038), indicating minimal evidence of inflation of significance of association findings (quantile-quantile plots are presented in Figure S1).

**Figure 1:**
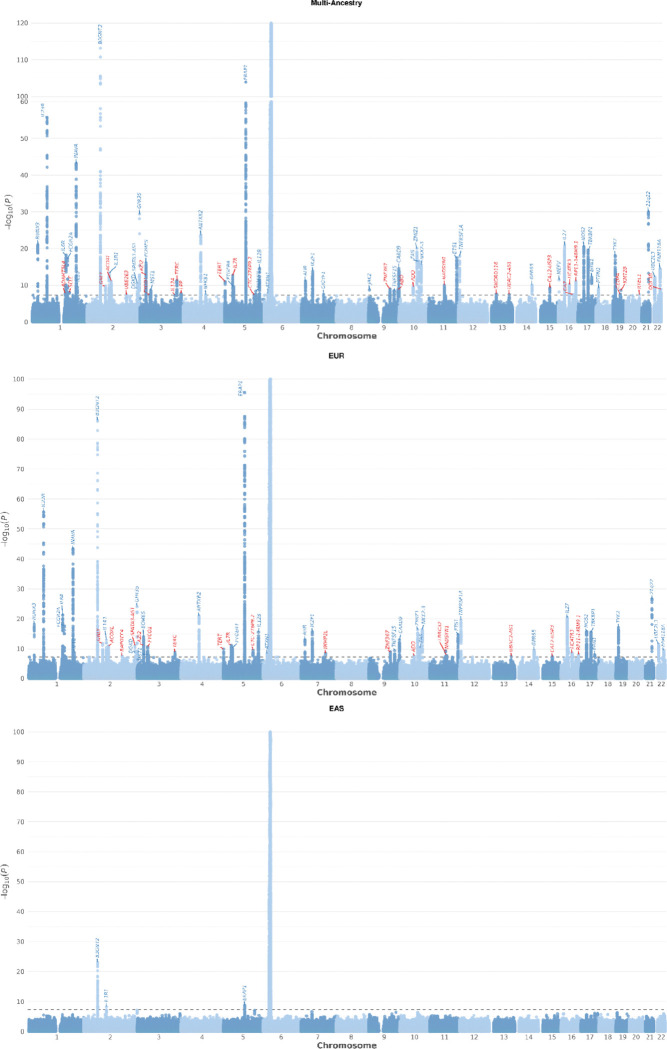
Manhattan plots for AS case-control GWAS in combined multiancestry, EUR and EAS analyses. The red horizontal line indicates the P = 5 × 10^−8^ genome-wide significant threshold. The blue horizontal line indicates the P = 10^−5^ threshold for ‘suggestive’ associations. Named genes are listed in blue for previously reported loci and red for novel loci. The y-axis has been abbreviated for each figure as per the legend.

**Figure 2:**
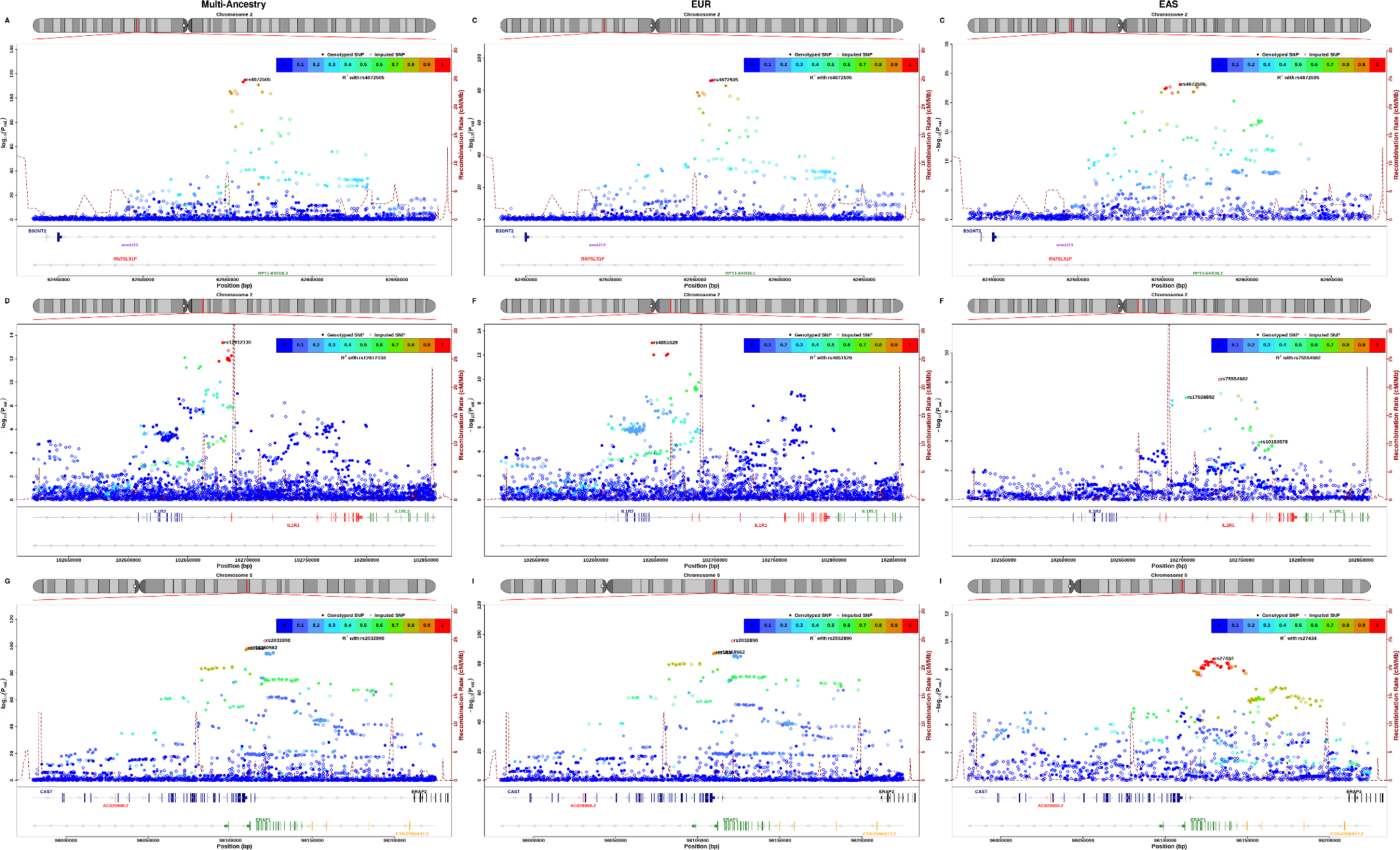
Locus association plots and linkage disequilibrium patterns with lead SNP for comparison of non-MHC associations identified at GWS across multiancestry (left column), EUR (middle column) and EAS (right column) analyses.

Overall, the study identified 69 non-MHC loci at GWS (Fig. 1, Table S2). Of these, 42 have previously been reported (Table S2) and 27 are novel (Table 1). Zoom plots for all associated loci are provided in Figure S2. All were found in the EUR ancestry participants excepting *MEFV* (association only present in Turkish cohort). In the EAS group association was observed with 3 known non-MHC loci, two previously also identified in EAS (chromosome 2p15, *ERAP1*) and one previously only reported in EUR-ancestry studies (*IL1R1*)(Fig. 2). Considering a less stringent threshold for previously reported loci: 47 previously reported non-MHC associations were also seen in the current study at 5 × 10^−8^ < P < 10^−5^ (‘suggestive’ association) and 96 at P < 0.05 (‘nominal’ association) (Table S3). Of 62 SNPs previously associated in an analysis of pleiotropic associations^18^, where the association was not GWS but showed experiment-wise significance for AS after controlling for other diseases, all 53 loci for which SNPs were available in this GWAS achieved P < 0.05, 30 achieved P < 10^−5^, and 9 achieved P < 5×10^−8^, providing further support that these loci are independently associated with AS (Table S3).

Imputed HLA-B associations were consistent with previous reports, with 82% of EUR cases were HLA-B27 positive compared with 8.6% of controls (Table S4). Apart from the well-established interaction between *HLA-B27* and *ERAP1* variants ^10,16^, no other genetic variant demonstrated statistically significant interaction with *HLA-B27* in our genome-wide gene-gene interaction analysis (Figure S3). Considering potential differences in genetic associations between males and females, the *HLA-B27* allele frequency was higher in males than females, consistent with previous reports (odds ratio (OR) = 1.43, P = 2.07×10^−8^). No non-MHC SNP demonstrated significant difference in strength of association between genders (P < 10^−5^).

### Independent Signals and Credible Causal Variants

Multi-ancestry fine-mapping was performed using SuSiEx ^19^. Of 69 non-MHC loci achieving GWS in the multi-ancestry analysis, independent signals were identified in 11, providing a total of 28 independent associations (Table S5). Thus, in total this study identified 86 independent non-MHC genetic associations with AS.

Considering the number of SNPs credibly associated with AS at individual loci, for 11 loci the 95% credible set was restricted to a single SNP, and for 30 independent signals was restricted to ≤ 5 SNPs. Three missense variants were identified amongst the 11 loci were a single SNP was involved. rs61752717 (M694I) lies in *MEFV* is strongly associated with increased AS risk in the Turkish cohort (OR = 5.26, P = 3.67×10^−12^), it being non-polymorphic in other ancestries. It is a known causative SNP for the monogenic autoinflammatory condition, Familial Mediterranean Fever. rs76418789 (G149R, OR = 0.73, P = 3.25×10^−8^) lies in *IL23R*. Its rare protective 149-R allele has been shown to lead to retention of the receptor in the endoplasmic reticulum and reduced IL-23 signalling and IL-17 production ^20^. rs34536443 (P1104A, OR = 0.73, P = 8.71×10^−19^) lies within *TYK2*, and the AS-protective 1104-A allele is a hypomorph ^21^.

### Heritability and Co-heritability analyses

The heritability explained by non-MHC common variants was similar between EUR and EAS cohorts after conversion to the liability scale. The liability-scale heritability was 0.17 (s.e. = 0.02) for EUR (assuming AS prevalence of 0.55%) and 0.11 (s.e. = 0.02) for EAS (AS prevalence 0.55%) (Table S6). Linkage disequilibrium score regression (LDSC) also indicated minimal confounding in both GWAS datasets, with LDSC intercept values of 1.05 for both EUR and EAS cohorts.

AS is clinically strongly correlated with inflammatory bowel disease (IBD) and psoriasis; and strong overlap of the genetic risk for these conditions has been previously reported ^18,22^. Our analysis confirmed strong coheritability of AS with IBD overall (rg = 0.55, P = 4.26×10^−36^) and with both ulcerative colitis (rg = 0.50, P = 3.17×10^−27^) and Crohn’s disease (rg = 0.50, P = 1.88×10^−23^) individually (Table S7). Coheritability with psoriasis was also observed (rg = 0.28, P = 1.98×10^−7^), though weaker than with IBD.

AS is also known to be associated with osteoporosis ^23^, even in early disease ^24^. We observed coheritability of the individual term osteoporosis (rg = 0.21, P = 2.30×10^−6^) and the combined term ‘osteoporosis, osteopenia, and/or pathological fracture’ (rg = 0.25, P = 2.35×10^−8^).

### Proteome and Transcriptome Analyses

To assist identification of key genes and pathways through which the observed genetic associations contribute to AS pathogenesis, we imputed serum protein and peripheral blood RNA profiles using the OmicsPRED platform and performed case-control analyses using the subset of EUR ancestry cases and controls genotyped with the Illumina Core-Exome SNP microarray (Table S8). To investigate protein levels directly in AS cases and healthy controls, serum protein levels were measured for 250 products (NULISAseq Inflammation Panel ^25^, ALAMAR Biosciences) in 298 cases and 142 healthy controls (Table S9). Additionally, we used this dataset and proteomic and transcriptomic data from the OmicPred Server to perform MR studies to explore potential causal relationships with AS.

Considering direct measurements: 70 proteins were differentially abundant in AS cases and healthy controls (FDR < 0.05). Of these, PWAS findings were available for 54, of which 19 proteins were imputed to be differentially abundant in cases and controls (P < 0.05), of which 9 were in the same direction in both direct measurements and imputed levels. These include B3GNT2, CCL2, CX3CL1, CXCL10, IL-12B, IL-2RB, MICA and TEK (decreased in cases in both direct measurement and imputed analyses), and CCL28 (increased in cases in both direct measurement and imputed analyses).

PWAS association was seen with 225 individual proteins in the overall dataset (FDR < 0.05). In the TWAS (RNA) analysis, 419 associations were identified (FDR < 0.05) including many AS-associated genes and relevant cytokines.

Considering cytokines and related gene products: increased imputed levels of IL-10, IL-11RA, IL-17F, IL-23R, IL-34, IL-36A, and IL-37 and reduced levels of IL-12A/B, IL-12B, IL-21, IL-27, IL-27RA, IL-6, and IL-6R proteins were identified (FDR < 0.05). Increased imputed levels of *IL2RA, IL2RB, IL23R, IL6ST, IRF5 and JAK2* mRNA were found in AS cases, and reduced mRNA levels of cytokine receptor genes *IL7R* and *IL1RL1*. Of these, GWS associations have previously been identified between AS and *IL23R* ^13^, *IL12B*, *IL21*, *IL6R* ^16^, *IRF5* ^18^, and in the current study, *IL7R*. Notably, IL-17 blockade and JAK inhibition are effective treatments for AS, consistent with these associations of genetically determined increased protein and/or mRNA with the disease.

AS cases were also imputed to have increased levels of *ERAP1* mRNA and protein, and increased levels of *ERAP2* RNA, consistent with previous studies which have demonstrated that AS risk-associated variants at both *ERAP1* and *ERAP2* increase transcription and translation of these genes ^26,27^. Considering other genes at loci associated with AS at GWS, *CMC1, DNMT3A, ERN1, GPR25, GPR35*, were imputed to have reduced RNA levels in AS cases, whereas imputed levels of *AHR, ANKRD55, ANTXR2, CARD9, FAM118A, FAS, IKZF1, IRF5, NOD2/CARD15*, and *TNFRSF1A* were increased in cases (FDR < 0.05). *ACOXL* encoded at novel GWS loci in the current study, was imputed to have increased mRNA levels in AS, consistent with a gain-of-expression mechanism underpinning these genetic associations.

### Cytokine-targeted therapies and AS

Genetic associations in genes or pathways targeted by therapies are predictive of the efficacy of those therapies, such as TNF and IL-17 targeted therapies in AS ^28^. Despite this and increased expression of these genes supporting their potential as treatment targets, unexpectedly trials of blockade of IL-6^29,30^, IL-12p40 (encoded by *IL12B*) (targeting IL-12 and IL-23) ^31^, and IL-23p19 (encoded by *IL23A*) (targeting IL-23) ^32^ did not display efficacy in AS. Increased gene or protein expression may result either from pathogenic mechanisms driving disease or as a secondary consequence of the disease itself, in which case it may not contribute to disease risk or persistence. In contrast, associations identified through PWAS, and TWAS reflect inherited genetic predisposition to disease onset and chronicity. As these genetic signals are fixed and independent of disease progression or activity, they offer a means to distinguish causal therapeutic targets from downstream effects of disease that may not be effective targets. Mendelian randomization (MR) provides complementary evidence by helping to differentiate causal effects from associations driven by secondary or reverse causation.

Measured serum levels of the proinflammatory cytokine IL-6 were increased in cases (vs. controls) in the current study (fold-change = 1.07, P = 8.70×10^−8^) consistent with previous reports^33^. IL-6 signals either directly (cis-signalling) through membrane-bound IL-6R, or indirectly (trans-signalling) through IL-6 binding soluble IL-6R including in cells that do not express membrane-bound IL-6R. IL-6ST/gp130 is a signal transducer involved in signalling not only from IL-6 but also from IL-27 and OSM (oncostatin M). IL-27 is an IL-12 family member with both pro- and anti-inflammatory functions, increasing production of the anti-inflammatory cytokine IL-10, and inhibiting Th17 responses, a key cell in AS inflammation. OSM is an IL-6 family member with proinflammatory and tissue repair functions. This study indicated that AS cases are genetically predisposed to have lower IL-6 and IL-6R levels (PWAS, FDR = 6.2×10^−8^ and 4.36×10^−7^ respectively), though higher IL-6ST levels (TWAS, FDR = 1.22×10^−5^). MR analysis indicates lower IL-6 protein levels are associated with risk of AS (FDR = 6.22×10^−8^). Genetic association was seen with SNP rs2228145 (IL-6R Asp358Ala, OR = 0.90, effect allele ‘C’, P = 5.09×10^−18^ combined dataset). This SNP allele is associated with increased shedding of membrane bound IL-6R and leads to a reduction in classical IL-6R signalling. This association suggests that reduced classical IL-6 signalling in particular is involved in protection from AS. These findings potentially explain why IL-6 blockade was ineffective in AS.

The locus encoding *OSM* was associated at GWS levels, PWAS demonstrated increased OSM in AS cases (P = 0.042), and measured serum OSM levels were marginally increased in cases (fold change 1.01, P = 0.043). OSM levels are elevated in AS patients, including in the sacroiliac joints ^34^. IL-27 protein levels were imputed to be reduced in AS cases in the current PWAS. Although previous reports have shown increased IL-27 in cases ^35^, no difference in measured serum levels between cases and controls was observed in the current study (P > 0.05). No trials of either OSM blockade or IL-27 treatment have been reported in AS. Consistent with our imputed findings, IL-27 treatment prior to disease onset in the HLA-B27-transgenic rat spondyloarthritis model inhibited spondyloarthritis development through suppression of IL-17/TNF production by CD4 + T-cells ^36^.

Considering IL-12, measured protein levels were reduced in cases (fold-change 0.99, P = 0.0034), as were imputed protein levels (FDR = 0.0033). For IL-23, whilst measured levels were increased in cases (fold-change = 1.02, P = 0.0061), imputed IL-23 levels were reduced in cases (FDR = 6.71×10^−8^). These findings may explain why IL-12 and IL-23 blockade were ineffective in AS.

### Intergenic Associations

**Figure 3:**
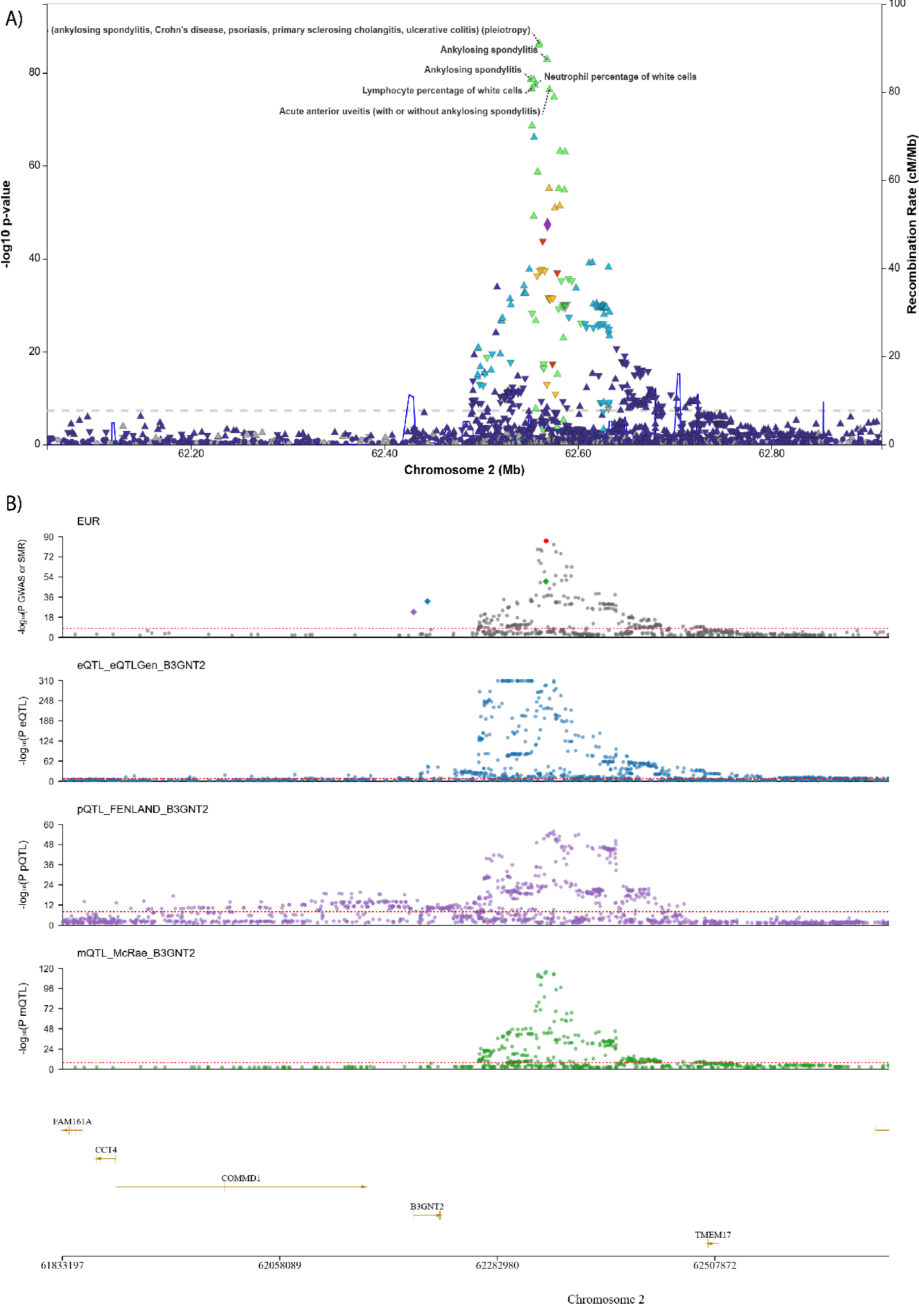
MR and GWAS catologue findings at chromosome 2p15 locus. a. GWAS Catalog associations at chromosome 2p15 in relation to SNP findings in EUR in the current study. b. SMR findings at chromosome 2p15, in relation to *B3GNT2*. Each y-axis represents −log10(P), the top row for the EUR GWAS, second row for eQTL, third row for Pqtl, and third row for mQLT. The red dotted line represents the significance threshold for the SMR test (P<8.4×10^−6^)

We previously reported associations at intergenic regions in chromosomes 2p15 and 21q22 ^15^. Considering the chromosome 2p15 association, using 2SMR, we found evidence to support *B3GNT2* (Beta-1,3-N-Acetylglucosaminyltransferase) as an AS-causative gene at chromosome 2p15. B3GNT2 influences T-cell activity through effects on glycosylation of costimulatory molecules. Several AS-risk SNPs at this locus are associated with immune-mediated inflammatory diseases including inflammatory bowel disease, primary sclerosing cholangitis and anterior uveitis, and peripheral blood neutrophil and lymphocyte cell counts (Fig. 3A). Summary-based-results MR analyses show pleiotropic association of AS-risk SNPs with *B3GNT2* RNA, protein, and methylation variation (Fig. 3B). Reduced levels of *B3GNT2* mRNA were causally associated with increased risk of AS (b=−0.250, P = 3.20×10^−13^)(Figure S4). Imputed *B3GNT2* RNA levels were lower in cases than controls (FDR = 1.68×10^−22^), as were imputed serum protein levels (P = 0.031). Directly measured B3GNT2 levels were also lower in cases than controls (P = 0.0076). The risk allele of the lead SNP at chromosome 2p15, rs4672505, is an eQTL for *B3GNT2* (GTEX, whole blood normalised effect size 0.19, P = 1.07×10^−29^), the risk A allele being associated with reduced *B3GNT2* transcription.

### ABO/FUT2/microbiome

We have previously reported association of *FUT2* variants in people of EUR ancestry^18^, and extend that observation here to include people of EAS ancestry (Table S10). *FUT2* encodes galactoside 2-L-fucosyltransferase, involved in glycosylation of ABO blood group antigens conferring their secretory properties across mucosal surfaces (‘secretor status’), which in turn influences the gut microbiome through effects on the gut mucin layer. Different SNPs determine secretor status in EUR and EAS ancestries (rs6013338 and rs1047781 respectively), with marked differences in population minor allele frequencies between ancestries. In the current study non-secretor status in EUR cases and controls, determined by homozygosity of rs6013338-A, increases the risk of AS (OR = 1.13, P = 1.11×10^−7^). The same direction of association was observed in EAS cases and controls, with non-secretor status determined by homozygosity of rs1047781-T also increasing risk of AS (OR = 1.21, P = 3.59×10^−7^).

Association of SNPs in the region of the ABO blood group locus was observed in the current study (rs687286, risk allele A, OR = 1.07, P = 1.67×10^−9^). Imputation of blood group antigens revealed increased AS risk with blood type A amongst EUR cases (OR = 1.064, P = 0.0059)(Table S11). No interaction was observed between secretor status, imputed blood group, and risk of developing AS.

We used MR analysis to investigate potential causal mechanisms between these associations and gut microbiome composition. For the exposure instruments, we obtained publicly-available data for GWS microbe QTLs ^37^. Briefly, this study performed GWAS on a meta-analysed population of EUR host gut microbiomes determined by 16S ribosomal sequencing. The microbial relative abundance quantitative analysis (mbQTL) contained 211 taxa. We identified a significant (FDR = 0.011) causal relationship between *Ruminococcus torques* and AS and non-secretor status, with reduced carriage associated with AS cases compared with HLA-B27-matched controls, consistent with previous studies ^38^.

### Telomere Length and AS

Lastly, we identified genetic association involving three genes involved in telomere maintenance (*TERT*, *TERC* and *RTEL1*). The strongest associated SNPs at *RTEL1* (rs71325458) and *TERT* (rs4449583) are eQTLs for each gene respectively (GTEX; rs71325458 for *RTEL1* expression, P = 1.59×10^−7^; rs4449583, for *TERT* expression, P = 3.12×10^−19^)(Figure S5) with the AS-risk allele for both genes leading to increased expression of the respective gene. To investigate these associations further we performed MR analysis of leukocyte telomere length (LTL) and AS (Figure S6, Table S12). We found that longer leukocyte telomere length was associated with increased AS risk (IVW: OR = 1.31, 95% CI: 1.11–1.54, P = 0.0012). The robustness of this finding was supported by sensitivity analyses, as the weighted median, and MR-Egger methods both yielded consistent results. MR-Egger regression indicated no significant directional pleiotropy (intercept = −0.0045, P = 0.27). The reverse MR analysis consistently showed no significant causal effect of AS on LTL across all five methods, with the IVW method yielding an OR of 1.00 (95% CI: 0.99–1.00, P = 0.12). The Gene Ontology enrichment analysis showed significant associations with telomere-related biological processes, particularly regulation of telomere maintenance via telomere lengthening (P_adjusted = 2.25×10^−8^) and negative regulation of telomere maintenance (P_adjusted = 5.21×10^−8^) (Table S13).

## Discussion

We report here the largest GWAS meta-analysis to date in AS, a common and disabling arthritis affecting approximately 1 in 200 individuals world-wide. Our study, involving 25,645 cases and 71,224 controls, identifies 27 novel loci and 86 independent genetic associations with AS Fine-mapping approaches identify key credible genetic variants at eleven of these loci and reduce the 95% credible set of SNPs to five or fewer in 31 loci. These provide strong additional evidence that the gut microbiome is critical to the pathogenesis of AS, as well as providing other novel findings regarding the disease’s pathogenesis. Through TWAS and PWAS studies we identify 419 associations with RNA species and AS, and 225 protein associations. These include multiple cytokine genes, genes with known immunological functions, and genes found at or nearby GWS loci. Lastly, our data may provide a genetic explanation as to why some treatment approaches have not been successful in AS, whilst also suggesting novel biological pathways for future therapeutic innovation.

The failures of IL-6, IL-12, and IL-23 blockade as treatments for AS were unexpected and, until now, remained unexplained. In the current study we demonstrate that genetic risk of AS is associated with reduced IL-6 levels (by both PWAS and 2SMR), whereas variable effects were seen on other IL-6 pathway and family members. This indicates that increased serum IL-6 levels in AS cases (seen in the current and previous studies) are not due to underlying genetic predisposition, rather are likely secondary to the disease itself. The reduction in imputed IL-6 and IL-6R suggests that reduction in IL-6 signalling increases AS risk. The AS-protective association of rs2228145 (IL-6Rasp358Ala), which leads to increased cleavage of membrane bound IL-6R, suggests that reduction in classical IL-6 signalling via membrane bound IL-6R is primarily involved in reduction in AS risk. These findings potentially explain why treatment with IL-6R blockade with tocilizumab was not effective in AS. Similarly, the genetic risk of AS was associated with reduced imputed IL-12A/B and IL-12B protein levels, which may explain the lack of clinical efficacy of ustekinumab (anti-IL-12B) in axial spondyloarthritis patients ^31^. No association was seen between imputed IL-23 protein levels and risk of AS, potentially explaining the lack of efficacy of IL-23 blockade in disease ^32^. In contrast, imputed levels of IL-23R protein and RNA were increased in AS cases; and inhibition of IL-17, which production is regulated by IL-23R, is highly effective in AS ^39^. Our findings thus highlight the value of genetic analyses in assessing potential therapeutic targets, including allowing discrimination between primary risk factors for a disease and secondary effects from the disease process itself.

In addition to providing a genetic explanation for the failure of specific cytokine inhibition therapies in AS, potential therapeutic candidates are identified. Credible set fine-mapping analysis in the current study demonstrates that the association of *TYK2* with AS is driven by the SNP rs34536443, a hypomorphic variant. TYK2 is a tyrosine kinase that mediates signaling through the type 1 IFN (IFNAR), IL-10 family (IL-10R and IL-22R), and IL-12 family (IL-12R and IL-23R) receptors. Several genes in the IL-10 and IL-12 family pathway are genetically associated with AS. The TYK2 inhibitor deucravacitinib has shown efficacy in treatment of psoriatic arthritis ^40^, but no trials have yet been reported in axial spondyloarthritis. In the SKG murine model of spondyloarthritis, TYK2 inhibition was shown to reduce spondyloarthritis severity ^41^. Our data provides further support for TYK2 as a therapeutic target in AS.

Considerable evidence suggests that AS occurs in genetically-predisposed hosts exposed to a common environmental agent(s). Reactive arthritis, a condition also strongly associated with HLA-B27, occurs following infection with a narrow range of gut and urogenital pathogens; subsequently, approximately 10% of affected individuals develop AS - which suggests that microbial agents are involved also in AS disease pathogenesis. This is supported by microbiome profiling studies which consistently demonstrate dysbiosis in AS cases ^42–45^. HLA-B27 carriage in healthy individuals without AS is also associated with gut dysbiosis, suggesting that these findings are related to genetically-influenced immune effects rather than purely due to effects of disease itself or its treatment ^38,46^. We have previously demonstrated that AS-associated genetic loci disproportionately involve genes expressed in gut tissue ^47^. In the current study we confirm association of *FUT2* with AS, with loss-of-function (‘non-secretor’) variants associated with increased AS risk. *FUT2* determines the ability to secrete ABO antigens across mucosal surfaces, and is associated with the gut microbiome. We extend this observation to demonstrate that AS is associated with ABO blood group carriage, with carriers of blood group A at increased AS risk. ABO antigens, in secretors, are expressed on gut mucins which are key gut barrier components. Mucins are large heavily-glycosylated proteins, and group A mucins have N-acetylgalactosamine at their terminal position which influences mucin viscosity and protective function, as well as favouring colonisation of microbes that prefer GalNAc-rich mucins such as certain Bacteroides or Faecalibacterium species ^48^. Lastly, through MR analysis, we demonstrate that non-secretor status causally contributes to AS pathogenesis via reduced carriage of *Ruminococcus torques*. In the general population, non-secretor genotype is strongly associated with increased carriage of Bifidobacteria and lower carriage of Ruminococcus species ^49^, and ABO blood group has been associated with carriage of Bifidobacteria ^50^. We have previously demonstrated increased carriage of Bifidobacteria and reduced carriage of Ruminococcus species in HLA-B27-positive AS cases compared with HLA-B27-positive healthy controls ^38^. Ruminococcus carriage also correlates inversely with disease activity in AS cases ^51^. Studies of the individual Ruminococcus species *Ruminococcus gnavus* have yielded conflicting findings, with both increased ^44^ and decreased ^52^ carriage in AS cases. In combination, these data support the gut microbiome as directly involved in AS pathogenesis; and, further, suggest that *Ruminococcus torques* carriage is associated with protection against the disease. *Ruminococcus torques* carriage is also increased in patients with IBD ^53^, and is mucolytic, both reducing the gut mucosal barrier integrity and influencing carriage of other bacteria dependent on mucin-derived nutrients ^54^. Reduced carriage of Ruminococcus may thus influence AS risk indirectly through effects on carriage of other gut bacteria. However, the gut microbiomes in AS and IBD cases are distinct ^46^; and it is notable that whilst terminal ileitis is common in AS it is typically subclinical, in contrast to Crohn’s disease. Reduced carriage of *Ruminococcus torques* in AS may protect AS cases with ileitis from developing clinical mucosal-eroding gut inflammation, through reduced in mucin degradation (in comparison with patients with Crohn’s disease). Further research into gut mucosal immunity and mucosal-adherent (rather than luminal) gut microbiota will be required to dissect this relationship further.

We identified novel associations in three genes (*TERT*, *TERC* and *RTEL1*) encoding components of the telomerase complex, involved in maintaining telomere length. Studies measuring telomere length have reported both increased ^55^ and reduced ^56^ length amongst AS cases. MR studies reported association of increased telomere length with AS (in both studies using AS case data from FinnGen in analyses) ^57,58^. We confirm these findings in the current study with increased telomere length was associated with increased risk of AS. Telomere length variation has multiple effects on immunological function including immune senescence, and production of cytokines including IL-1β, and TNF-α ^59^, and long leukocyte telomere length has been associated with increased risk of SLE ^57^. Association of variants near *RTEL1* have been previously reported with IBD ^60,61^. Loss of function mutations in *TERC* are associated with hemophagocytic lymphohistiocytosis, a severe systemic hyperinflammatory syndrome ^62^. Further research will be required to determine the mechanisms underlying the increased telomere length, and how that influences the immune system to increase AS risk.

This study provides strong evidence that the intergenic region at chromosome 2p15 operates to influence AS risk via effects on *B3GNT2.* This locus is also associated with psoriatic arthritis ^63^, IBD ^64^, acute anterior uveitis ^65^, and IgA nephropathy ^66^, each of which are clinically associated with AS. The association of this locus with AS has been widely replicated in different ancestries ^67–69^. B3GNT2 mRNA levels negatively correlate with markers of AS-disease activity (ESR, CRP) and extent of syndesmophyte development ^69^. This is consistent with our findings that genetic risk variants at this associated locus operate through reduction of B3GNT2 RNA and protein levels to increase disease risk; and that in AS cases serum B3GNT levels are reduced ^69^. B3GNT3 elongates cell surface long-chain poly-N-acetyl-lactosamine, leading to suppression of immune responses; and B3GNT2−/− mice develop T- and B-cell hyperactivity and elevation of proinflammatory cytokine levels including TNFa and IL-1b ^70^. This may be due to reduced polyactosamine on costimulatory molecules such as CD19 and CD28, with polylactosamine levels on these molecules in B3gnt2−/− mice lower than in wild-type mice ^70^. In tumour models, B3GNT2 over-expression increases resistance to T-cell mediated cytotoxicity ^71^. These findings point to a new pathway involved in the pathogenesis of AS and multiple related diseases sharing genetic association with the chromosome 2p15 intergenic region.

AS is associated with osteoporosis, likely for multifactorial reasons including disease-associated inflammation, elevated cytokine levels, immobility, and/or glucocorticoid treatment. In the current study, significant genetic covariance between osteoporosis and AS suggests that a genetic risk of osteoporosis is associated with increased risk of AS. Bone mineral density is significantly genetically determined (h2 = 30–70% at lumbar spine and hip) ^72,73^. It is not unexpected that shared genetic factors would be involved between osteoporosis and AS, given the clinical association between the two conditions, even in early AS ^74^. This further emphasizes the need to screen AS patients for osteoporosis, even amongst those with controlled inflammation and who have not received glucocorticoids. AS is localised to sites of bone stress, initially in the sub-fibrocartilaginous bone of the sacroiliac joints and also later the peri-entheseal bone in the spine ^75^. This raises the possibility that skeletal biomechanical stress could have a more detrimental effect and potentially be linked to disease onset in subjects with lower bone density.

In conclusion, this study identifies 27 novel loci and 86 independent genetic associations with AS, along with over 600 genes implicated by TWAS and PWAS as involved in the pathogenesis of the disease. These findings point to potential new therapeutic targets and provide genetic explanations for the failure of previous clinical trials of inhibition of major cytokine pathways in AS. Our findings further highlight the key role of the gut microbiome in AS pathogenesis, and identify novel pathways in the disease including those involving telomere length and costimulation inhibition through genetic effects on polyactosamine deposition. These findings advance our understanding of the pathogenesis of AS and point to novel therapeutic development for the disease.

## Methods

### Participants

Before quality control, we included 26,113 AS cases and 72,793 control individuals of EUR, EAS, Turkish, and Iranian ancestry cohorts (Table S1). AS was defined according to the 1984 modified New York criteria ^76^. All cohorts obtained informed consent from all participants following protocols approved by their institutional ethical committees. The overall approval was given by Queensland University of Technology Health Research Ethics Committee (approval number HREC/05/QPAH/221).

### Genotyping

The study cohort comprised 96,686 participants who underwent genotyping using various platforms. Specifically, 50,278 individuals were genotyped using the Illumina CoreExome chip, 20,032 using the Illumina Global Screen Array, and 1,683 using the Illumina OmniExpress Array. Additionally, 20,214 participants were genotyped with the Illumina Immunochip platform, while the remaining 4,462 individuals were assessed using the Illumina OmniZhonghua array.

### Data quality control

Quality control (QC) was performed in individual chip type in each ancestral group. We first excluded, for each collection separately, SNPs with call rate < 95% or with a Hardy-Weinberg equilibrium (HWE) P value < 1×10^−6^ in controls, and samples with call rate < 95%. For overlapped SNPs across cohorts, we performed pairwise missingness tests between the collections and removed all SNPs with differential missingness (P < 1 × 10^−7^). We further removed samples with extreme heterozygosity (threshold > 3 standard deviations from mean). Relatedness was assessed in each chip type/cohort separately, by calculating identity by descent using PLINK (v1.90, https://www.cog-genomics.org/plink/1.9/) ^77^. For each pair of related samples (PI_HAT > 0.2), the sample with the lower call rate was removed, and, where the pair involved a case and a control with similar call rates, cases were preferentially selected for inclusion.

We then confirmed origins of samples of EUR, Asian, Iranian and Turkish ancestry by principal-component analysis (PCA). Genotype data were merged with genotype information from seven HapMap 3 populations (CEU, TSI, YRI, MEX, JPT, CHD and CHB), and samples were identified as ethnic and ancestry outliers (more than 6 standard deviations from the mean of principal component of the relevant HapMap ancestry).

After QC, 25,645 cases and 71,224 controls remained for analysis.

### Imputation

After QC, genotyping data for EUR, EAS, TK and IR were imputed using 1000 Genomes Project Phase 3 through the Sanger Imputation Service (www.sanger.ac.uk/tool/sanger-imputation-service/). We applied stringent post-imputation filters, excluding variants with an imputation quality score (INFO) ≤ 0.6. Additionally, standard QC metrics were implemented, including HWE testing (P < 1×10^−6^), SNP missingness rate (< 5%), and minor allele count ≥ 20 thresholds.

HLA imputation was then performed using the Michigan Imputation Server, with the Four-digit Multi-ethnic HLA reference panel v2. The imputation was done for the classical HLA genes, variants, and presence/absence of specific amino acid residues. Imputed alleles were retained for downstream analysis with imputation quality score (R^2^) > 0.7. Post-imputation QC steps included removing variants with call rates < 95%, HWE P < 1×10^−6^, and MAF < 0.5%.

### Association and meta-analysis

Chip-type and ancestry-specific association analyses were performed using logistic regression in PLINK v2 software (https://www.cog-genomics.org/plink/2.0/), with the first 10 principal components included as covariates to control for population stratification. The ancestry-specific and overall meta-analysis was performed by inverse-variance method implemented in the software package OSCA v 0.46.1. All conducted statistical tests were two-sided, with GWS level of P < 5 × 10^−8^ and genome-wide suggestive level at 5 × 10^−8^ < P < 1×10^−5^.

### Heritability and genetic correlation analysis

We estimated SNP-based heritability (h^2^) for EUR and EAS populations separately using LDSC (V1.0.1). Heritability estimates were transformed to the liability scale using a prevalence of AS of 0.55% in both populations. As LDSC requires large GWAS sample sizes, and is best suited for ancestrally homogeneous cohorts, this analysis was limited to the EUR and EAS GWAS. Ancestry matched linkage disequilibrium reference panels were used.

Genetic correlation (rg) between AS and different phenotypes was calculated using LDSC as implemented in the https://vl.genoma.io/webserver ^78^. Only GWAS summary statistics from EUR ancestry were analyzed, since most GWAS datasets in CTG-VL are derived from EUR cohorts. GWAS summary statistics were filtered to include SNPs with MAF > 1% and excluded MHC region.

### Omicspred imputation

Imputation of protein abundance was performed using pre-trained prediction weight files from the OmicsPred resource. Briefly, post-QC imputed genotype data were used to infer protein or molecular abundance by applying a weighted sum of dosage genotypes, with effect sizes specified in the OmicsPred weight file for each molecule. The weighted sum was calculated using PLINK v1.9, based on the provided dosage genotypes and corresponding effect sizes.

### ABO groups and Secretor status imputation

The ABO gene, located at chromosome 9q34.2, encodes a glycosyltransferase enzyme responsible for transferring nucleotide sugars to the H antigen, thereby generating the ABO blood group antigens. The four main ABO blood groups (A, B, AB, and O) can be differentiated using three key single nucleotide polymorphisms (SNPs) within this gene: rs8176719 (c.261delG), rs8176746 (c.796C > A), and rs8176747 (c.803G > C) ^79,80^. The 261delG deletion (rs8176719) identifies the O allele, while rs8176746 and rs8176747 distinguish between the A and B alleles. ABO blood group phenotypes were determined from these genetic variants (Table S10).

Secretor status was inferred from genotype data at population-specific *FUT2* polymorphisms, following established conventions. The specific SNPs and corresponding genotype definitions were selected based on individual ancestry (Table S11).

We performed a multivariable logistic regression analysis to assess the associations between ABO group, secretor phenotype, and sex with the outcome phenotype (AS vs. non-AS) among all European participants, excluding the Immunochip cohort due to missing marker SNPs required for ABO blood group imputation. Specifically, we fitted a logistic regression model including all main effects and all possible two-way and three-way interactions among ABO group, secretor phenotype, and sex:

phenotype~ABOgroup×secretorstatus×sex

All variables were included as categorical predictors. The model was implemented in R using the glm function with a binomial family and logit link.

where *phenotype* is the binary outcome variable (0 = control, 1 = case); *ABO group* is a categorical predictor variable, representing the individual's ABO blood group (e.g., A, B, AB, O); *secretor status* is encoded as non-secretor status and secretor status. All variables were included as categorical predictors. The model was fitted using the glm function in R (version 4.3.3) with a binomial family and logit link. Statistical significance was set at p < 0.05.

### Interaction with HLA-B27

To investigate potential interactions between imputed HLA-B27 status and GWS loci, we tested all 69 lead SNPs identified from the primary EUR meta-analysis. For each SNP, an interaction term between HLA-B27 and the SNP was included in the regression model:

phenotype~SNP+SEX+B27+B27*SNP+B27*SEX+PC1+PC2+…+PC10

where phenotype is the quantitative phenotype; SNP is genotype dosage (0, 1 and 2) for the tested lead SNP of the locus; SEX is sex (binary); B27 is HLA-B27 status (carrier/non-carrier); B27*SNP is the interaction between B27 and lead SNP of the locus; B27*SEX is the interaction between B27 and sex; and PC1-PC10 are the first ten principal components for population structure adjustment.

### Independent signals with credible sets of causal variants

For each risk region associated with AS, we performed multi-ancestry fine-mapping using the Bayesian approach implemented in SuSiEx (v1.1.0). Population-specific LD reference panels for EUR, EAS, Turkish, and Iranian ancestries were constructed from CoreExome Array genotyping data. Risk loci were defined as a ± 250kb region around each lead variant. For each independent signal within a risk region, we derived a 95% credible set of candidate causal variants (CCVs). The variant with highest posterior inclusion probability (PIP) within the credible set was designated as the lead variant for that signal. Risk region signals were excluded if their credible set did not contain any variant with a marginal P value < 1 × 10⁻^5^ in any ancestry-specific meta-analysis.

### Mendelian randomization studies

To prioritize potential causal genes at AS-associated loci, we integrated GWAS findings with multi-omics data using summary-based Mendelian randomization (SMR). The SMR analyses assessed the effects of genetic variants on gene expression using blood eQTL data from eQTLGen and GTEx v8, splicing events using sQTL data from GTEx v8, blood DNA methylation using mQTL data from McRae et al. ^81^, and plasma protein abundance using pQTL data from the SCALLOP consortium (www/scallop-consortium.com). Lead variants were also queried in Open Targets Genetics (https://genetics.opentargets.org), and for each locus the gene with the highest overall Variant-to-Gene (V2G) score was selected as the most likely candidate gene.

Given the association of SNPs near three telomere maintenance genes (*RTEL-1*, *TERC*, and *TERT*) with AS, we conducted a 2SMR analysis using the ‘*TwoSampleMR*’ package in R v 4.4.1 to investigate the potential causal relationship between leukocyte telomere length (LTL) and AS. LTL (n = 472,174) was obtained from the UK Biobank genome-wide association study pooled data ^82^. Genetic variants associated with telomere length at GWS were selected as instrumental variables from UK Biobank summary statistics, with variants located in the major histocompatibility complex (MHC) region excluded. AS summary statistics were derived from our own EUR ancestry meta-analysis, after filtering out variants with MAF < 1%. In order to avoid weak instrument bias, SNPs with F-statistics less than 10 were excluded. Exposure and outcome datasets were harmonized to align effect alleles. Outliers and potential horizontally pleiotropic variants were identified and removed using MR-PRESSO. The inverse-variance weighted (IVW) method was used as the primary MR analysis. Further sensitivity analyses were performed, including MR-Egger regression, weighted median estimation, and leave-one-out analysis, to evaluate the robustness of our findings and assess potential pleiotropy.

MR analyses of the potential causal role of microbiome components and AS were conducted using *R/Bioconductor* v 4.0.2 and the ‘*TwoSampleMR*’ package. Briefly, summary statistics from the EUR subset of the AS GWAS were used as the outcome data set. Publicly available summary statistics were used from a recent meta-analysis and GWAS of host microbiome quantitative trait loci (mbQTLs) ^37^. Each microbial taxon was defined as a single exposure set. All variants passing a pre-defined threshold of P < 10^−5^ for each exposure taxa were used to ensure the maximum number of overlapping loci between the exposure and outcome data sets. Following initial analysis, MR causality tests were performed using the Wald test, and Wald ratios were meta-analysed using inverse weighted variance regression, as previously performed to ensure consistency of results across studies ^83,84^.

### Proteome Study

Protein level analysis was performed using *R/Bioconductor* v 4.0.2 and included a modified protocol ^85^. The ‘*NULISAseqR*’ package specifically developed by Alamar Biosciences for their data formats was used for analyses. Briefly, attomolar concentrations of panel proteins were adjusted for limit of detection controls per plate and uploaded into R in a matrix format using the ‘*readNULISAseq*’ function. Based on the assumption that most proteins would not be differentially expressed between cases and controls across a panel of proteins, we used TMM normalisation (primarily associated with RNAseq data analysis) on the raw protein concentration data to normalise the matrix prior to protein case control analysis. Following this initial correction, a model matrix was constructed with case and control information and possible confounding variables (batch number, patient ID, age at collection, smoking status, drug treatments). The ‘*voom*’ function within the limma package was used to normalise each protein in the array data according to the model matrix ^86^. As some of the samples were longitudinal for the same AS patients, we used a paired design matrix to further normalise the quantity matrix and account for samples with the same patient ID. The normalised quantity matrix was fit to a linear model, and a contrast matrix between cases and controls was used to instruct the model to compare groups. Lastly, the normalised and contrasted matrix was fitted to a Bayesian model to gauge the magnitude of protein quantity differences. The final numbers of associated proteins between cases and controls were summarised using the ‘*decideTests’* function in limma. Multiple testing was addressed by correcting raw P-values using an FDR < 0.05.

## Supplementary Material

Supplementary Files

This is a list of supplementary files associated with this preprint. Click to download.

• FigureS1.docx

• FigureS2.docx

• FigureS3.docx

• FigureS4.docx

• FigureS5.docx

• FigureS6.docx

• SupplementaryTables.xlsx

## Figures and Tables

**Table 1:** Novel associations in multi-ancestry GWAS meta-analyses at genome-wide significance (P<5×10^−8^).

CHR	Location	SNP rsID	Nearby gene	EA/NEA	EAS_EAF	EUR_EAF	OR	95%CI OR	P
1	150527294	rs12124948	*ADAMTSL4*	T/C	0.442	0.414	1.08	1.05–1.11	1.59×10^−8^
1	169728137	rs2420505	*SELE*	G/A	0.477	0.191	1.08	1.05–1.11	8.63×10^−9^
2	85933367	rs6547632	*GNLY*	A/G	0.384	0.391	0.93	0.90–0.95	1.56×10^−10^
2	111811665	rs4848370	*ACOXL*	C/T	0.482	0.276	0.92	0.90–0.94	7.99×10^−12^
2	182038445	rs12620692	*UBE2E3*	G/T	0.352	0.420	0.93	0.91–0.95	3.92×10^−8^
2	231839516	rs3106083	*GPR55*	G/A	0.043	0.396	1.11	1.08–1.14	2.99×10^−11^
3	45995708	rs532777636	*FYCO1*	TGG/T	NA	0.103	0.79	0.73–0.86	3.30×10^−8^
3	159637678	rs76830965	*IL12A*	A/C	0.001	0.114	0.89	0.85–0.93	4.94×10^−8^
3	169486508	rs35446936	*TERC*	G/A	0.456	0.241	1.11	1.08–1.14	5.68×10^−12^
3	188125120	rs13093110	*LPP*	C/T	0.386	0.455	0.94	0.92–0.96	1.74×10^−8^
5	1284135	rs4449583	*TERT*	T/C	0.381	0.334	1.09	1.06–1.11	1.04×10^−11^
5	35879156	rs6881706	*IL7R*	T/G	0.177	0.266	0.91	0.88–0.93	2.18×10^−13^
5	134451317	rs17659254	*CTC-276P9.2*	G/C	0.196	0.332	1.07	1.04–1.10	4.13×10^−8^
9	99172627	rs10118598	*ZNF367*	T/C	0.014	0.174	1.11	1.07–1.15	1.03×10^−9^
9	136137106	rs687289	*ABO*	A/G	0.438	0.330	1.07	1.05–1.10	1.67×10^−9^
10	64503349	rs224063	*ADO*	T/G	0.400	0.411	0.93	0.91–0.95	2.33×10^−10^
11	71159764	rs28364617	*NADSYN1*	G/T	0.445	0.236	0.92	0.90–0.94	9.33×10^−11^
13	40536069	rs9576932	*SNORD116*	A/T	0.361	0.436	1.08	1.05–1.11	2.54×10^−8^
13	99809175	rs2390194	*UBAC2-AS1*	A/G	0.373	0.468	1.07	1.04–1.09	3.50×10^−8^
15	63789952	rs17765311	*CA12-USP3*	C/A	0.005	0.355	1.1	1.07–1.14	4.63×10^−10^
16	50166602	rs11076514	*HEATR3*	G/T	0.312	0.242	0.92	0.89–0.94	6.59×10^−11^
16	66894332	rs35301918	*CA7*	T/A	0.396	0.446	0.94	0.92–0.96	3.66×10^−8^
16	80044738	rs9935292	*RP11-148M9.1*	T/C	0.437	0.422	0.92	0.90–0.95	8.15×10^−12^
19	33741951	rs34635674	*CEBPA*	G/A	0.031	0.332	0.93	0.90–0.95	2.62×10^−8^
19	36213072	rs28373672	*KMT2B*	G/A	0.207	0.235	1.1	1.06–1.13	2.47×10^−9^
20	62268333	rs71325458	*RTEL1*	G/A	NA	0.019	0.72	0.64–0.81	3.81×10^−8^
22	50971047	rs361725	*ODF3B*	T/C	0.273	0.371	1.08	1.05–1.10	1.18×10^−9^

CHR, chromosome; Location, extracted from human genome assembly GrCH38; EA, effect allele; NEA, non-effect allele; EAF effect allele frequency; EAS, east Asian ancestry; EUR, European ancestry; OR, odds ratio, P P value.
